# Interaction modifications lead to greater robustness than pairwise non‐trophic effects in food webs

**DOI:** 10.1111/1365-2656.13057

**Published:** 2019-08-11

**Authors:** J. Christopher D. Terry, Rebecca J. Morris, Michael B. Bonsall

**Affiliations:** ^1^ Department of Zoology University of Oxford Oxford UK; ^2^ School of Biological Sciences University of Southampton Southampton UK; ^3^ St. Peter's College Oxford UK

**Keywords:** ecological network, food web, higher‐order effect, non‐trophic effect, robustness, stability, trophic interaction modification

## Abstract

1. Considerable emphasis has been placed recently on the importance of incorporating non‐trophic effects into our understanding of ecological networks. Interaction modifications are well‐established as generating strong non‐trophic impacts by modulating the strength of interspecific interactions.
2. For simplicity and comparison with direct interactions within a network context, the consequences of interaction modifications have often been described as direct pairwise interactions. The consequences of this assumption have not been examined in non‐equilibrium settings where unexpected consequences of interaction modifications are most likely.
3. To test the distinct dynamic nature of these “higher‐order” effects, we directly compare, using dynamic simulations, the robustness to extinctions under perturbation of systems where interaction modifications are either explicitly modelled or represented by corresponding equivalent pairwise non‐trophic interactions.
4. Full, multi‐species representations of interaction modifications resulted in a greater robustness to extinctions compared to equivalent pairwise effects. Explanations for this increased stability despite apparent greater dynamic complexity can be found in additional routes for dynamic feedbacks. Furthermore, interaction modifications changed the relative vulnerability of species to extinction from those trophically connected close to the perturbed species towards those receiving a large number of modifications.
5. Future empirical and theoretical research into non‐trophic effects should distinguish interaction modifications from direct pairwise effects in order to maximize information about the system dynamics. Interaction modifications have the potential to shift expectations of species vulnerability based exclusively on trophic networks.

## Graphical abstract

Non‐trophic interactions are increasingly thought to be important, but provide complex challenges. Here the authors show that the higher‐order nature of interaction modifications is important to understand their impact through generalised food web simulations.

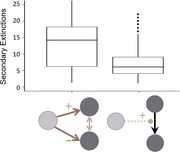

## 1 INTRODUCTION

There is a building appreciation that to improve our understanding of population dynamics within ecological communities, it is necessary to move beyond studies that focus on a single interaction process at a time (Kéfi et al., 2012; Levine, Bascompte, Adler, & Allesina, 2017). Trophic interaction modifications (TIMs) (Terry, Morris, & Bonsall, 2017; Wootton, 1993) occur when a consumer–resource interaction is modulated by additional species. These are a class of higher‐order processes since their effects are not fundamentally pairwise. Examples include associational defences (Barbosa et al., 2009), fear effects (Sih, Englund, & Wooster, 1998), certain impacts of ecosystem engineers (Sanders et al., 2014) and foraging choices (Abrams, 2010). It has been empirically demonstrated that many strong non‐trophic effects (NTEs) are caused by such processes, with large implications for community structure and dynamics (Ohgushi, Schmitz, & Holt, 2012; Preisser, Bolnick, & Benard, 2005; Werner & Peacor, 2003). Furthermore, it has been repeatedly shown that interaction modifications can cause qualitatively distinct responses to perturbations than may otherwise be expected (Barbosa, Fernandes, Lewis, & Morris, 2017; Donohue et al., 2017; Matassa, Ewanchuk, & Trussell, 2018; van Veen, van Holland, & Godfray, 2005).

Approaches to understanding interaction modifications often try to distil the inherently multi‐species process into a pairwise NTE (or “trait‐mediated indirect effect”) from the modifier species onto one or both recipient species (Okuyama & Bolker, 2007, Figure 1). This allows the direct comparison of non‐trophic and trophic interaction strengths (Preisser et al., 2005) and network structure (Pilosof, Porter, Pascual, & Kéfi, 2017) but is a representation of a different class of dynamic process (Terry et al., 2017). This simplification can give valuable insights into communities at equilibrium (e.g. Grilli, Barabás, Michalska‐Smith, & Allesina, 2017). However, the consequences of this assumption in a transient, fluctuating or heavily perturbed system have yet to be fully explored. Previous studies introducing interaction modifications to trophic networks (Arditi, Michalski, & Hirzel, 2005; Goudard & Loreau, 2008; Lin & Sutherland, 2014) have demonstrated their potential impact on the dynamics of ecosystems, but not whether this is attributable to the higher‐order nature of interaction modifications, as opposed to shifts in connectance and interaction strength.

An important case is the dynamics of ecological systems in the face of species removal, where there is the potential for secondary extinctions and eventually the collapse of the ecosystem (Dunne, Williams, & Martinez, 2002). This aspect of stability, often described as “robustness,” is important both from the perspective of managing anthropogenic change and in terms of understanding the fundamental stability of ecological communities. Since empirically testing how whole communities respond to extinctions can be difficult or impossible (although see Sanders, Thébault, Kehoe, and Frank van Veen (2018)), a number of studies have attempted to determine the properties that make ecological communities robust through simulation (Dunne & Williams, 2009; Säterberg, Sellman, & Ebenman, 2013). However, incorporating the acknowledged flexibility of ecological networks is a perennial challenge for such studies (Montoya, Pimm, & Solé, 2006).

The impact on the robustness of ecological networks of one specific subset of interaction modifications, those caused by flexible foraging in response to resource availability, has been examined in a number of studies (Valdovinos, Ramos‐Jiliberto, Garay‐Narváez, Urbani, & Dunne, 2010). Approaches have included topological rewiring (Gilljam, Curtsdotter, & Ebenman, 2015; Kaiser‐Bunbury, Muff, Memmott, Müller, & Caflisch, 2010; Staniczenko, Lewis, Jones, & Reed‐Tsochas, 2010; Thierry et al., 2011), multi‐species functional responses (Uchida & Drossel, 2007) and adaptive foraging models (Kondoh, 2003). These models showed that the additional dynamic process impacted robustness in contrasting directions, but only addressed a restricted subset of interaction modifications caused by predator switching. However, consumption rates are influenced by more than just the choice of prey available to the consumer. Interaction modifications can also be caused by threats to the consumer (Suraci, Clinchy, Dill, Roberts, & Zanette, 2016), facilitation (Bruno, Stachowicz, & Bertness, 2003), associational susceptibility (Underwood, Inouye, & Hambäck, 2014) or mutualistic defence (Holland, Ness, Boyle, & Bronstein, 2005), amongst others (Ohgushi et al., 2012). This introduces a considerable number of additional links between species in ecological communities, yet studies of generic interaction modifications within large networks are limited (Arditi et al., 2005; Bairey, Kelsic, & Kishony, 2016; Garay‐Narváez & Ramos‐Jiliberto, 2009; Goudard & Loreau, 2008; Lin & Sutherland, 2014), with most theoretical analyses of interaction modifications focussing on small community units (Abrams, 2010; Bolker, Holyoak, Křivan, Rowe, & Schmitz, 2003; Holt & Barfield, 2013).

Calls to incorporate the full panoply of NTEs into our understanding of ecological networks have built substantially in recent years (Fontaine et al., 2011; Ings et al., 2009; Kéfi et al., 2012; Levine et al., 2017; Ohgushi et al., 2012; Olff et al., 2009), and the first empirical inventories are being established (Kéfi et al., 2015; Kéfi, Miele, Wieters, Navarrete, & Berlow, 2016). Theoretical analyses can play a significant role in motivating the empirical construction of networks, identifying the information necessary to best understand these systems. Here, we demonstrate the distinctive nature of interaction modifications compared to pairwise NTEs through a direct standardized comparison of their impacts on the robustness of large artificial networks. We then examine the role of interaction modifications in determining species‐level vulnerability to secondary extinction.

## 2 MATERIALS AND METHODS

We conducted our analyses using model food webs where system dynamics are derived from metabolic scaling relationships. As detailed below, interaction modifications were introduced to a set of communities each at an initial equilibrium. Robustness to extinction was examined by introducing external mortality to a single species at a time and integrating the model to a new equilibrium. All analyses were carried out in r v.3.5.0 (R Core Team, 2018) using the *deSolve* numerical integration package (Soetaert, Petzoldt, & Setzer, 2010), and all code and data are available online.

### 2.1 Bio‐energetic model

The change in biomass density, $B_{i}$, of each species in the community was modelled using a simple Lotka–Volterra type model with a linear (Holling type I) functional response and logistic intrinsic growth rates, parameterized using body‐mass relationships:

(1) $$\frac{dB_{i}}{\mathrm{dt}}=B_{i}\left(r_{i}-m_{i}-\frac{B_{i}}{K_{i}}-\sum_{j\;\in \;\text{consumers}\;\text{of}\;i}\mu_{\mathrm{ij}}a_{\mathrm{ij}}B_{j}+\sum_{l\;\in \;\text{resources}\;\text{of}\;i}\mu_{\mathrm{li}}e_{\mathrm{li}}a_{\mathrm{li}}B_{l}\right)$$

Each species was assigned a body mass ($M_{i}$) drawn from a distribution based on their trophic level (see Appendix S1: Section S1) which was then used to calculate further parameters using quarter‐power body‐mass scaling laws (Yodzis & Innes, 1992). Relative intrinsic growth or metabolic loss rates, $r_{i}$, were set at 1 for all producers (trophic level 1) and $r_{i}=-0.1M_{i}^{-0.25}$ for each consumer (trophic level ≥ 2). Consumer‐specific consumption rates were set at $a_{\mathrm{ij}}=\omega_{j}M_{j}^{-0.25}$, where the generality term $\omega_{j}$ was fixed at 1/n, the number of resources of each consumer j. Assimilation efficiencies, $e_{\mathrm{ij}}$, for each trophic interaction were drawn from a uniform distribution centred around 0.1 (Moore & de Ruiter, 2012), $e_{\mathrm{ij}}∼U\left(0.05,0.15\right)$. Carrying capacities, $K_{i}$, were drawn from $K_{i}∼U\left(1,10\right)$ for producers (to introduce a moderate degree of self‐regulation) and $K_{i}∼10^{U\left(2,3\right)}$ for consumers (considerably higher than the starting populations and so introducing only a small amount of self‐regulation). Initially, external mortality, $m_{i}$, was set to 0 and modification terms, $\mu_{\mathrm{ij}}$, set to 1.

The trophic topology and population densities of a set of 200 starting communities for the robustness tests were generated as follows. Initial trophic topologies were generated using the niche model (Williams & Martinez, 2000), with 35 species and a connectance of 0.14, removing cannibalistic interactions. Each population density was initially set to 10 and the system numerically integrated to a stable equilibrium (see Appendix S1: Section S4 for criteria). Only fully connected communities with at least 18 persisting species were retained. Properties of the starting communities are described in Appendix S1: Section S2.

### 2.2 Specification of TIMs

Trophic interaction modifications were introduced through modification terms, $\mu_{\mathrm{ijk}},$ that specify the impact of modifier species k on the consumption of species i by species j, where i, j and k are all different. These are positive numbers that multiply the attack rate as a function (detailed in the next section) of the divergence of the biomass density of a modifying species, $B_{k}$, from its starting equilibrium value $B_{k}^{*}$. Consequently, when $\mu_{\mathrm{ijk}}$ is smaller than 1, the interaction is weakened and when $\mu_{\mathrm{ijk}}$ is greater than 1, the interaction is strengthened. Where multiple species modify the same interaction, these effects were assumed to be synergistic and combine multiplicatively, $\mu_{\mathrm{ij}}=\prod^{k}\mu_{\mathrm{ijk}}$ (Golubski & Abrams, 2011; Goudard & Loreau, 2008). In our model, $\mu_{\mathrm{ijk}}$ cannot be negative, preventing the reversal of the direction of the trophic interaction.

**Figure 1:**
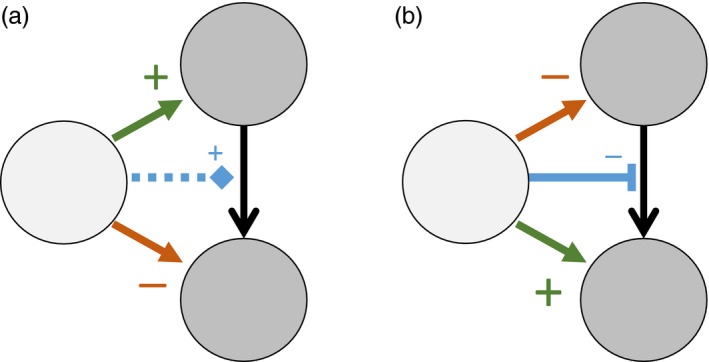
Depiction of distinction between trophic interaction modifications (TIM) and consequent non‐trophic effects (NTEs) when describing the impact of a modifier species on a consumer–resource pair. In (a) the modifier species causes a facilitating TIM (blue dashed line) where an increase in the modifier leads to a beneficial NTE on the consumer and a detrimental NTE on the resource. Correspondingly, in (b) an interfering TIM causes a beneficial NTE on the resource and a detrimental NTE on the consumer

Interaction modifications cause both positive and negative NTEs (Figure 1). Where an increase in the modifier species leads to an increase in the strength of the interaction, we describe the modification as a *facilitating* TIM. This can be said to be beneficial to the consumer and detrimental to the resource, in the sense of the immediate impact from an increase in the modifier population. We term the reverse situation an *interfering* TIM.

### 2.3 Functional form of TIM

**Figure 2:**
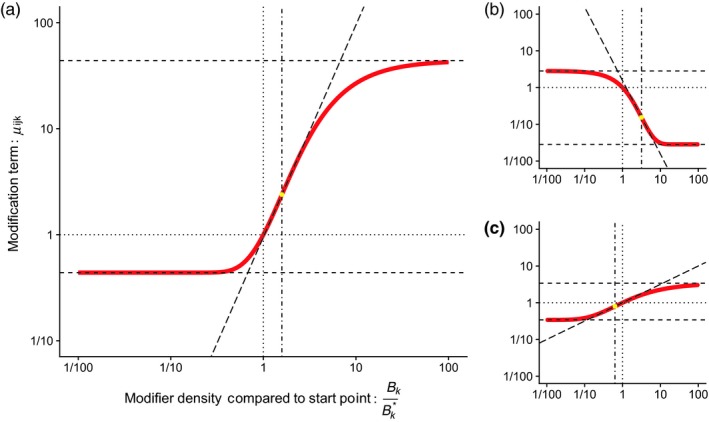
Graphical representation of the relationship between the control parameters and the response of the sigmoidal function used to determine the modification term $\mu_{\mathrm{ijk}}$ from the density of the modifier $B_{k}$. Panel (a) uses a maximum slope (*α*) of 2, a distance to threshold (*τ*) of 0.2 and a maximum magnitude of difference (*σ*) of 2. This describes a facilitatory modification where an increase in the modifier population increases the strength of the modified interaction. The greatest rate of change occurs slightly above the starting point, and the modification is 100× as strong when the modifier is highly abundant compared to when it is very rare. Panel (b): *α* = −2, *τ* = 0.5, *σ* = 2, shows an interfering modification where an increase in the modifier leads to a decline in the interaction strength. Here the interaction can be proportionally reduced much more than it can increase. Panel (c): *α* = 0.5, *τ* = −0.2, *σ* = 1 shows a weaker facilitatory modification, where the threshold has been exceeded. Note that the function is calculated on a base 10 logarithmic scale

To represent the relationship between the density of the modifier species and the modification of the interaction, we used a Gompertz sigmoidal curve parameterized to control features of ecological relevance, detailed in full in Appendix S1: Section S3. This function links the magnitude of divergence of $B_{k}$ from its start point $B_{k}^{*}$, as $\log_{10}\left(B_{k}/B_{k}^{*}\right)$, and three control parameters (Figure 2). These are α (the maximum rate of change in the modified interaction as the modifier density increases), τ (the proportional change from $B_{k}^{*}$ to reach the threshold point of maximum response) and σ (the range of magnitudes over which $\mu_{\mathrm{ijk}}$ spans). A positive *α* denotes a facilitatory modification and a negative *α* an interfering modification. A key attribute of our parameterization is that when $B_{k}=B_{k}^{*},\mu_{\mathrm{ijk}}=1$. This maintains the original trophic interaction strength when the modifier is at its starting equilibrium point—in effect, these strengths are assumed to already incorporate the effect of the modifier to the interaction at the equilibrium.

### 2.4 Alternative representation of TIMs as pairwise non‐trophic effects

**Figure 3:**
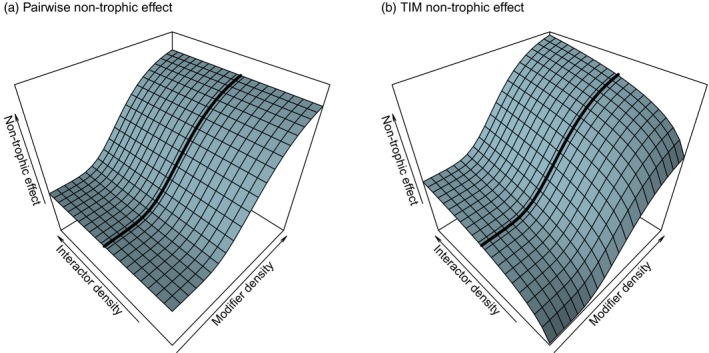
Illustrative response surfaces showing the distinction between non‐trophic effects caused by pairwise interactions (a) and those from a higher‐order interaction modification process involving three species (b). Per capita effects are shown in the vertical axis on a logarithmic scale. In both cases, the response to the modifier is identical at the starting density of the trophic interaction partner of the focal species, shown by the black line. However, the density of the trophic interaction partner of the focal species (left‐hand axis) does not affect the non‐trophic effect in the pairwise case (a), but does with the full interaction modification model (b). Note also that as the interactor's density becomes small, the strength of the non‐trophic effect rapidly declines

As an alternative to modelling the impact of TIMs in full (using “higher‐order” terms), equivalent pairwise effects can be derived. These match the impact of the full TIM model, the only distinction being the non‐trophic effect from a modifier k to a trophic interactor is no longer dependent on the biomass of the other member of the trophic pair (Figure 3). To maintain parity with the full TIM model, this was done by first partitioning the interaction term into trophic and non‐trophic components, then fixing the value of the other trophic interactor to the density at the original equilibrium, denoted $B^{*}$. Full steps of the derivation are detailed in Appendix S1: Section S6. A trophic interaction affecting a resource i influenced by a modifier k can be partitioned from:

(2) $$\frac{1}{B_{i}}\frac{dB_{i}}{\mathrm{dt}}=\ldots \underset{\text{Trophic Term with TIM}}{\underbrace{-\mu_{\mathrm{ijk}}a_{\mathrm{ij}}B_{j}}}$$

to:

(3) $$\frac{1}{B_{i}}\frac{dB_{i}}{\mathrm{dt}}=\ldots \underset{\text{Trophic}\;\text{Term}}{\underbrace{-a_{\mathrm{ij}}B_{j}}}+\underset{\text{Non-Trophic}\;\text{Term}}{\underbrace{a_{\mathrm{ij}}B_{j}^{*}\left(1-\mu_{\mathrm{ijk}}\right)}}$$

The corresponding terms for the interactions affecting the consumer, j, are as follows:

(4) $$\frac{1}{B_{j}}\frac{dB_{j}}{\mathrm{dt}}=\ldots +\underset{\text{Trophic}\;\text{Term}}{\underbrace{e_{\mathrm{ij}}a_{\mathrm{ij}}B_{i}}}+\underset{\text{Non - Trophic}\;\text{Term}}{\underbrace{e_{\mathrm{ij}}a_{\mathrm{ij}}B_{i}^{*}\left(\mu_{\mathrm{ijk}}-1\right)}}$$

As an example, a facilitating TIM, when $B_{k}$ increased, would lead to a $\mu_{\mathrm{ijk}}$ greater than 1. This would lead to a negative NTE on the resource i (independent of $B_{j}$) and a positive NTE on the consumer j (independent of $B_{i}$). This partitioning process (and hence direct comparison between full and pairwise models) is only straightforward when each trophic interaction is modified by at most one modifier species because of the synergistic relationship between multiple modifiers assumed by our model.

### 2.5 Test 1. Comparison of robustness between pairwise and higher‐order models of TIMs

To compare the consequences of introducing TIMs by these two approaches, we conducted three sets of robustness tests using the same set of trophic networks. The first used the full TIM model, the second used TIMs that had been converted to pairwise form and a third case without any TIMs.

We randomly added TIMs to the set of initial communities such that each potential modification (combination of trophic interaction and modifier species) had an equal 0.05 chance of existing. For these tests, each interaction was modified by at most one other species to allow the conversion to NTEs. Hence, in a community with s species, each interaction has a $0.05\times \left(s-2\right)$ chance of being modified and a typical community with 20 species and trophic connectance 0.14 would on average have 48 TIMs. Shape parameters for each TIM were drawn from uniform distributions: slope $\alpha ∼U\left(-4,4\right),$ range $\sigma ∼U\left(0.1,4\right)$ and threshold $\tau ∼U\left(-1,1\right)$. The location of TIMs and their shape parameters were identical between the full TIM and pairwise models.

For each robustness test, the external mortality rate ($m_{i}$) of a single species (which we will refer to as the “targeted” species for convenience, although the mortality could be attributable to a range of non‐directed processes) was then set to 1, the system numerically integrated to a new steady state and the status of each species in the resultant community assessed (Appendix S1: Section S4). Species were considered “extinct” if their biomass fell below $10^{-4}$ (several orders of magnitude below the starting values of most of the populations, Appendix S1: Section S2), “functionally extinct” if their biomass fell to below 1/10th of their starting value and considered to have “exploded” if the final density was over 10 times the starting value. Robustness tests were repeated, targeting each species in turn for each community, to give a total of 3,736 completed tests (93.8% successful integrations, Appendix S1: Section S4).

### 2.6 Test 2. TIMs and distribution of extinctions

To examine the relationship between the distribution of TIMs and consequent secondary extinctions, the robustness tests of the full TIM case as described above were repeated. For this test, a higher occurrence rate of TIMs (0.08) was used and the restriction that only one modification can affect each trophic interaction was relaxed. Results are reported for parameters drawn from $\alpha ∼U\left(-3,3\right),\sigma ∼U\left(0.1,3\right)$ and $\tau ∼U\left(-1,1\right)$. Further tests, with lower TIM occurrence rates and more restricted distributions of shape parameters, reached qualitatively similar results. Properties of the community and the relationship between the “targeted” species and the extinct species were then calculated.

Firstly, for each robustness test, the trophic distance from the targeted species to each secondarily extinct species was calculated. This is the number of trophic interactions between the targeted species and the secondarily extinct species by the shortest route using the starting network. To generate a baseline to compare against, the trophic distance from the targeted species to every other species was calculated.

Secondly, the NTEs affecting each extinct species at the start of the simulation were counted and classified by whether an increase in the modifier would initially be beneficial or detrimental for the focal species. This was calculated in two ways: firstly, counting all NTEs affecting the extinct species and secondly, only those derived from TIMs where the modifier species was the targeted species. In our model, species that are involved in a greater number of trophic interactions will tend to be the recipients of a greater number of NTEs. To distinguish the effect of increased trophic degree from the number of NTEs, for each species, we calculated the expected number of incoming NTEs given its trophic interactions and the number of potential modifiers to generate a baseline. The actual number of incoming NTEs of each category (beneficial or detrimental) for each extinct species was then compared to this expectation baseline.

## 3 RESULTS

**Figure 4:**
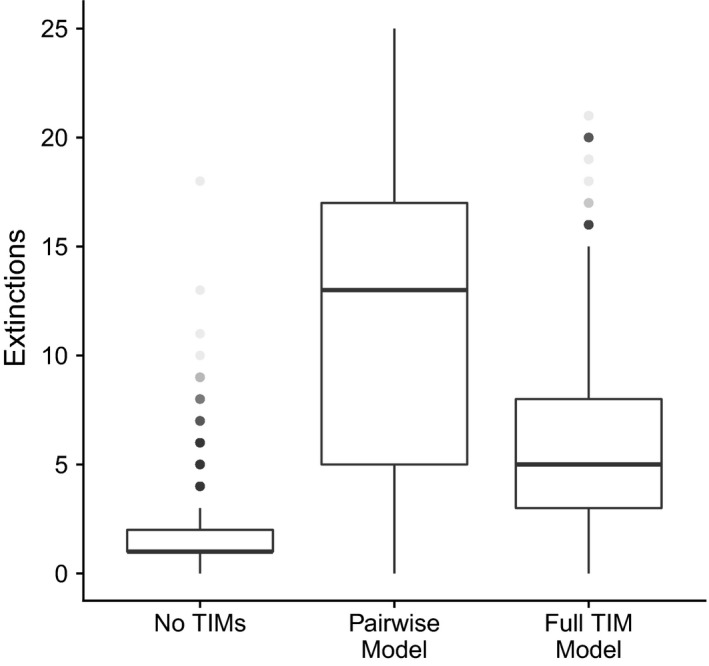
Boxplot showing that robustness was significantly lower when (TIMs) were modelled as pairwise interactions rather than directly as full interaction modifications. Both cases produced significantly more extinctions than the ‘No TIM’ case (paired *t* tests, *p* < .0001, *n* = 3,942)

The introduction of TIMs greatly increased the number of extinctions (Figure 4). However, almost double the number of extinctions were observed when the interaction modifications were represented with the pairwise model compared to when they were modelled directly with the full TIM model (*M*: 11.4 [*SD*: 6.76] against 5.74 [*SD*: 3.74], *t* test paired by community and targeted species ID: *p* < .0001, *t* = 61.5, *df* = 3,941). These results were very similar when functional extinctions were also included (*M*: no TIM: 1.98 [*SD*: 1.39], pairwise: 11.33 [*SD*: 6.75], full TIM: 5.85 [*SD*: 3.80]). Population explosions were rare in all scenarios. Without TIMs, there was a mean of 0.09 (*SD*: 0.32) explosions per robustness test, with on average nearly twice as many under a pairwise model (*M*: 0.189, *SD*: 0.529). The full TIM model resulted in the fewest explosions per robustness test (*M*: 0.05, *SD*: 0.239).

**Figure 5:**
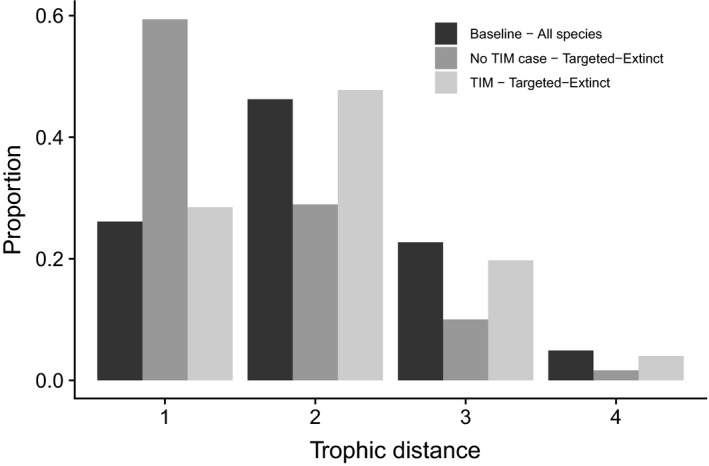
Distribution of trophic distances across the set of communities. Trophic interaction modifications shift the distribution of extinction vulnerability from nearby species towards an equal likelihood. Without TIMs, extinct species frequently interact directly with the targeted species (dark grey bars). With the inclusion of TIMs, the distribution of trophic distances between the targeted and the extinct species (light grey bars) closely follows the baseline distribution of distances between species (black bars). Trophic distances above 4 were very rare in our models and not shown

In robustness tests without TIMs, nearly 60% of secondary extinctions observed across all the communities were species directly trophically linked to the targeted species (Figure 5). The introduction of TIMs shifted the distribution of trophic distances (the number of trophic links between species by the shortest route) between target and secondarily extinct species towards the baseline distribution of trophic distances (Figure 5).

Secondarily extinct species tended to be recipients of both more beneficial (*M* = 4.53, *SD*: 1.52) and more detrimental (*M* = 4.02, *SD*: 1.53) NTEs than would be expected to affect an average species (*M* = 3.79, *SD*: 1.2, *t* tests paired by species ID, both *p* < .0001, *n* = 3,576). When counting just NTEs from the targeted species, secondarily extinct species tended to have more beneficial (*M* = 0.289, *SD*: 0.27) but fewer detrimental (*M* = 0.178, *SD*: 0.20) NTEs than the baseline expected number (*M* = 0.207, *SD*: 0.06, *t* tests paired by species ID, all *p* < .0001, *n* = 3,576).

**Figure 6:**
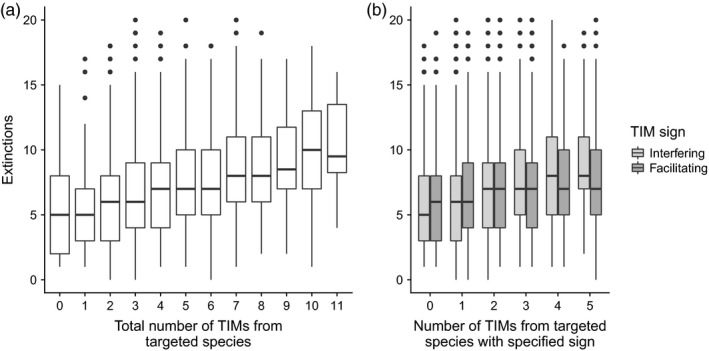
Boxplots showing the increase in the resultant number of extinctions as the number of trophic interaction modifications (TIMs) caused by the targeted species increased. Panel (a) shows the response to the total number of TIMs. Panel (b) shows the distinct responses to the number of interfering and facilitating TIMs

The targeting of species that caused more TIMs induced more extinctions (Figure 6a, Poisson glm, *p* < .0001, *n* = 3,825), with each additional TIM caused by the targeted species leading, on average, to an additional 7% secondary extinctions. Model fits splitting the sign of the TIMs (Figure 6b) indicated that each additional interference TIM caused a greater increase in the number of extinctions (+10.4%), compared to each additional facilitatory TIM (+4.1%). Including the trophic connectance of the web as an additional predictor variable did not significantly improve the model fit ($\chi^{2}$ test, *p* = .113).

## 4 DISCUSSION

Here, we have examined the ecological robustness of networks to demonstrate that interaction modifications have distinct dynamic effects. In ecological systems, efforts to include NTEs into food webs (Fontaine et al., 2011; Pilosof et al., 2017) should take into account the higher‐order nature of interaction modifications. Without quantitative information of the topological and strength distribution of TIMs, it is not yet possible to precisely calculate their impacts relative to trophic interactions. Nevertheless, it is clear that they have the potential to change our expectation of both the relative vulnerability of species to extinction and the impact the loss of certain species will have (Donohue et al., 2017).

We found that when TIMs were fully represented our model systems were notably more robust than under an exclusively pairwise model, yet the overall number of “direct” relationships between species through all types of interaction is the same between our two representations of TIMs. While higher trophic connectance can increase robustness to secondary extinctions (Dunne & Williams, 2009), increased connectance due to TIMs has the opposite effect in our model. However, when isolated from connectance, the higher‐order nature of trophic interactions increases robustness. This aligns with the results of Bairey et al. (2016) who found that higher‐order interactions could increase the persistence and local stability of unstructured interaction matrices. The quantification of interactions and connectance in higher‐order systems is a challenge (Golubski, Westlund, Vandermeer, & Pascual, 2016). Our results highlight the need to develop how the complexity of higher‐order systems can be best discussed and analysed, since differences between these models would not be picked up under classic ecological complexity measures (e.g. May, 1972).

In part, our result can be attributed to the tendency for an overall decline in population densities after perturbation (Appendix S1: Section S7). In the full TIM model, the strength (and disruptive influence) of a modification is dependent on the product of two species and so (in the context of generally declining species densities) will decline faster than when the strength is dependent on just one species. Weaker NTEs will reduce the potential for cascading effects from species removal, analogous to the impact of trophic link strength (Zhao et al., 2016). Furthermore, if the density of either consumer or resource falls to zero, any TIM impact on the species it is trophically linked to would also fall to zero under the full TIM model. However, with the pairwise model, the NTE is maintained even though the mechanism is no longer extant. Hence, as species become extinct, the effective connectance of the full TIM model declines faster than in the pairwise case. These mechanisms would only be evident in non‐equilibrial analyses.

There are also differences between the full TIM and the pairwise models in terms of the prevalence and rapidity of local feedback loops. Feedbacks modulating the impact of TIMs are heavily dependent on the relative speed and strength of multiple feedback loops, which in turn are derived from properties of individual species. Despite our relatively simple model, these loops are challenging to trace in large complex systems, limiting qualitative analysis (Dambacher & Ramos‐Jiliberto, 2007). Nonetheless, as the impact of the more complex full TIM model is dependent on more species, in general, it appears that mitigating feedback loops can be shorter and hence act faster in the full TIM case. For example, the effects caused by an interaction modification (under either model) will cause an immediate increase in one affected species and decrease in the other. In the full TIM model, where the impact of the interaction modification is dependent on $B_{i}\times B_{j}$, the strength of the impact of the TIM on the consumer and the resource will be initially mitigated since $B_{i}$ and $B_{j}$ move in opposite directions. In the pairwise model, the strength of the NTE on each species in the trophic pair will rapidly diverge. This has the potential to generate unrestricted positive effects on one of the populations, with disruptive effects for the rest of the community. Over longer time‐scales, changes in consumption rate lead to consistent changes in the equilibrium values of both consumer and resource. In a simple module, a heightened consumption rate leads to greater suppression of the resource which in turn can support a reduced population of consumers (e.g. Morin, 2011). These distinct phases of responses to a change in consumption rate, immediate biomass shifts in opposite directions before eventually moving towards a consistent direction, highlight the multiple time‐scales over which TIMs operate. The analysis of the contributing factors to these feedback loops would be a profitable, although challenging, area for future work.

At least in this simple model, it appears that TIMs disperse relative extinction risk from those species that are closely trophically connected to the perturbed species, to be more evenly distributed throughout the network. Trophic interaction modifications greatly increase network connectivity—for instance, our inclusion of randomly distributed TIMs in our second analysis brought the mean overall path length between species down from 3.0 to 1.6 and halved the mean network breadth from 4.2 to 2.1. While it is likely that in real ecological systems, many TIMs (for example, those caused by consumer‐avoidance responses) link trophically close species, others such as those caused by ecosystem engineer species may well create the long links that cause this short‐circuit effect.

Overall, the species that went extinct tended to be those that were affected by more TIMs. When considering NTEs from all species, this was true of both “beneficial” and “detrimental” NTEs. Those species that directly benefit from the presence of other species are clearly affected by extinction cascades. However, those species receiving an above‐expectation number of detrimental NTEs were also at higher risk of extinction despite the prospect that these species would benefit from the on‐average reduction or removal of their inhibitors. The correlation between the increased species‐level trophic connectance and the number of modifications received was accounted for by comparing expected number of NTEs on a per‐species basis. In part, the vulnerability of species subject to detrimental NTEs can be explained by temporary increases in modifier species. Initial responses to a small reduction in the population of a single species were as commonly positive as negative (51.1% of non‐zero growth‐rate responses to a 1% density reduction in a random species were positive in the TIM models, Appendix S1: Section S5). The low number of species maintaining large increases at the end of the process (what we defined as population explosions) shows that such increases were relatively transient. When considering only TIMs from the targeted species, which always decrease in density, extinct species received fewer than expected detrimental NTEs, supporting this direct explanation. Our results suggest that, although species facilitated by other species are indeed more sensitive to extinction, it is the overall number of relationships with other species that is critical. Both apparently “beneficial” and “detrimental” processes should be considered on an equal footing.

Much previous work has shown that complex networks are stabilized by consistent patterns in key parameters, which can be derived from body‐mass scaling rules (Brose, Williams, & Martinez, 2006; Otto, Rall, & Brose, 2007). In our model, this source of stability declines, as these patterns are disrupted by interaction modifications that effectively push each attack rate value out of the allometrically specified range in both directions. Tracking modification dynamics during simulations is complex, but it is clear that attack rates shifted considerably in our model. To take one illustration, the extinction of a modifier species in our second test would cause a median attack rate change of a factor of 5.62, but with a long tail of larger modifications. Attack rates in real systems take place in the context of other species, and significant disruptions to pairwise interaction strengths derived from laboratory experiments caused by interaction modifications have been empirically observed (Jonsson, Kaartinen, Jonsson, & Bommarco, 2018). The extent of attack rate and interaction strengths variability is an important qualifier to observed allometric patterns.

Our choice of a Gompertz function to represent interaction modifications, although mathematically complex in form, offers certain advantages compared to previous linear (Arditi et al., 2005; Bairey et al., 2016), exponential (Goudard & Loreau, 2008; Lin & Sutherland, 2014) or rational function (Sanders et al., 2014) models. In particular, it has the ability to directly and independently control salient features of the function with clear ecological relevance (distance to threshold, maximum rate of change, range between maximum and minimum). The dependence on the relative divergence from a particular starting point rather than the absolute value of the density of the modifier will often be more straightforward to compare to observational data where absolute values may be uncertain. Describing modifications in terms of changes to the original populations can be more directly related to pressures upon those populations.

Nevertheless, this is still a highly simplistic model. The interaction modifications included in this study were introduced at random, in the sense that each potential modification had an equal chance of existing. Considerable stabilizing structuring has been observed within a rocky shore NTE network (Kéfi et al., 2015; Kéfi, Miele, et al., 2016) but there is not yet a sufficient diversity of examples to be able to determine whether there are consistent features across ecosystems. The distribution interaction modifications, both in terms of strength and position relative to the trophic network, can lead to emergent structures that affect stability (Terry, Bonsall, & Morris, 2018). If interaction modifications are influential, results showing that stability can derive from structures observed in purely trophic networks such as trophic coherence, modularity and nestedness (Johnson, Domínguez‐García, Donetti, & Muñoz, 2014; Stouffer & Bascompte, 2011; Thébault & Fontaine, 2010) may need to be revisited (Levine et al., 2017).

Our model was highly linear, and there is considerable scope for further work accounting for the impact of ecological nonlinearities that can have interacting effects with higher‐order interactions (Letten & Stouffer, 2019; Sentis & Boukal, 2018). Firstly, saturating functional responses are near‐universal in ecological systems (Kalinkat et al., 2013) and may act to limit the potential impact of increases in trophic interaction rates mediated by TIMs by placing an upper limit on the overall intake rates. There is considerable scope to compare the impact of modifications to different aspects of nonlinear functional responses beyond overall consumption rate (Kéfi et al., 2012). Secondly, further work is needed to explore the consequences of nonlinear combination of multiple TIMs, beyond the synergistic assumptions used here. It is a reasonable estimate that many modification effects act antagonistically to each other (Golubski & Abrams, 2011). For instance, the presence of a second fear‐inducing predator may well have less effect than the first, dampening the impact of a change in either modifier population. However, the empirical base to parameterize any such analyses is currently very small.

Further opportunities for future work include introducing specific accounting for the time‐scale of changes and the size of perturbations. It is possible that higher‐order interactions are stabilizing against small perturbations, which may make the system as a whole more susceptible to large impacts, such as the extinction of certain species (Levine et al., 2017). Trophic interaction modifications also have the potential to create the necessary positive feedback structures to maintain alternative stable states (Holt & Barfield, 2013; Kéfi, Holmgren, & Scheffer, 2016). As yet, the prevalence of these features is largely unknown. The speed of interaction modifications themselves can also vary. While many TIMs are behaviourally mediated and occur essentially instantaneously, others are due to accumulated environmental changes (Sanders et al., 2014) or evolutionary processes (Benkman, Siepielski, & Smith, 2012) and operate at somewhat slower time‐scales.

## 5 CONCLUSIONS

In summary, interaction modifications are potent forces that introduce distinct dynamics to ecological networks. This distinctive nature of interaction modifications is of relevance for dynamic systems in many fields that make use of networks (Strogatz, 2001) since our work shows that the complexity of networks is more than the product of connectance and the number of interacting units. Despite long‐standing calls for the inclusion of NTEs into the mainstream of ecological network science that has been long dominated by food webs (Ings et al., 2009), and the publication of the first empirical community level non‐trophic network (Kéfi et al., 2015), there remains a great number of significant unknowns about the role of NTEs at the network scale. Our work shows that maintaining interaction modifications as distinct processes within empirical and theoretical networks, rather than as pairwise NTEs (Grilli et al., 2017), will enable a more complete understanding of the system dynamics and allow better predictions of community responses to perturbations. When documenting non‐trophic interactions, identifying processes as interaction modifications need not necessarily require significant additional effort on the part of the original investigator, but would be challenging for others to retroactively discern from published pairwise interaction networks. Analyses of network robustness are used extensively to understand anthropogenic impacts on natural communities (Evans, Pocock, & Memmott, 2013; Kaiser‐Bunbury et al., 2017; Säterberg et al., 2013); as the development and analysis of comprehensive interaction networks expand (Kéfi, Miele, et al., 2016), we must incorporate interaction modifications appropriately.

## AUTHORS' CONTRIBUTIONS

J.C.D.T. initiated the research; J.C.D.T., M.B.B. and R.J.M. contributed to the ideas presented in the manuscript; J.C.D.T. conducted the research, facilitated by discussions with R.J.M. and M.B.B.; J.C.D.T. wrote the manuscript; and all authors contributed to revisions and approved the final version.

## Data Availability

All R code used in this study and the generated simulation results are available on the Open Science Framework: https://doi.org/10.17605/OSF.IO/W83BR (Terry, 2019) or github.com/jcdterry/TIMs_and_Robustness.
