# Supplementary material for: Interaction modifications lead to greater robustness than pairwise non‐trophic effects in food webs
Source: J Anim Ecol. 2019 Aug 11;88(11):1732–42. doi: 10.1111/1365-2656.13057 (PMC6900167; doi:10.1111/1365-2656.13057)
Supplement: Supplementary file 1 [file JANE-88-1732-s001.docx]

**Supplementary Information**

Code and simulation data are available on the Open Science Framework repository at https://osf.io/w83br/DOI: 10.17605/OSF.IO/W83BR and github.com/jcdterry/TIMs_and_Robustness

**1. Method for determining body masses from trophic level**

Body masses are assigned based on trophic level in the original, pre-integration to equilibrium, communities. First, trophic levels calculated on using the pathway method of Levine (1980), rounded into integer trophic levels. These trophic levels were then used to assign relative body masses specified on a logarithmic scale (base 10). We used distributions with a lower variance than empirical distributions to aid the stability of our systems by distributing body-masses in a relatively hierarchical manner with trophic level.

1. Producers were assigned a body mass of 0.
2. Primary consumer (Trophic level 2)) body-masses were drawn from a normal distribution: $\mathcal{N}\left. \left( 0.65,\sigma=0.5 \right. \right)$, truncated at 0 to remove the possibility that consumers are smaller than herbivores.
3. Higher-level consumer (Tropic level 3+) body masses were specified based on the sum of repeated draws from a normal distribution for each trophic level above 2 (h = TL-2), standardising the variance

$$M_{i}= 0.65 + \sum_{1, \ldots, h} \mathcal{N}\left. \left( 2.73,\sigma=\frac{0.5}{\sqrt{h}} \right. \right)$$

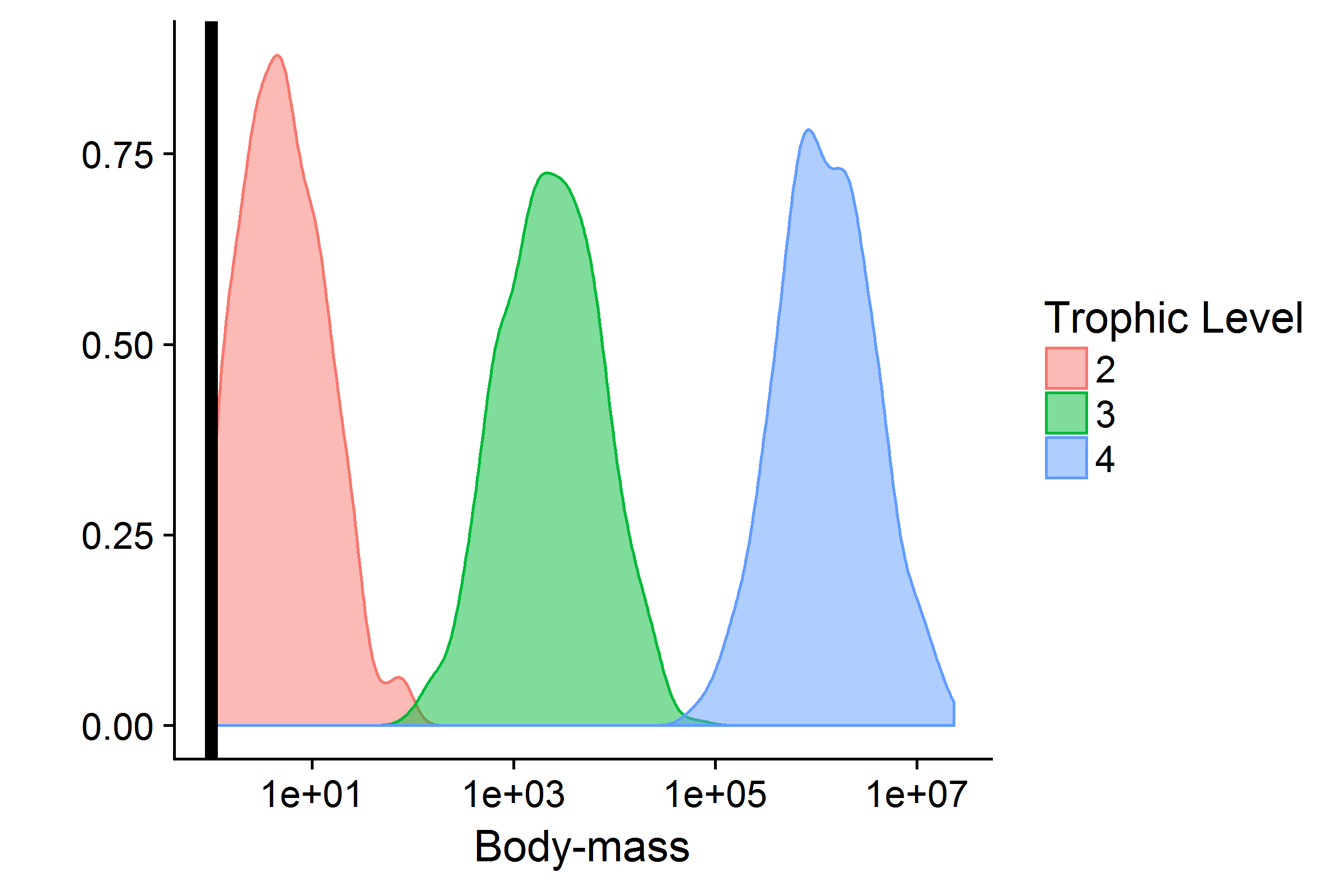


Figure S1. Density plot showing distribution of body masses across integer trophic levels. Black line shows producer body mass. Note the relatively small overlap in body masses and logarithmic scale.

**2. Information about starting populations**

The baseline communities are available in R list format as part of the online supplementary material. Summary statistics are shown in the Figure S2.


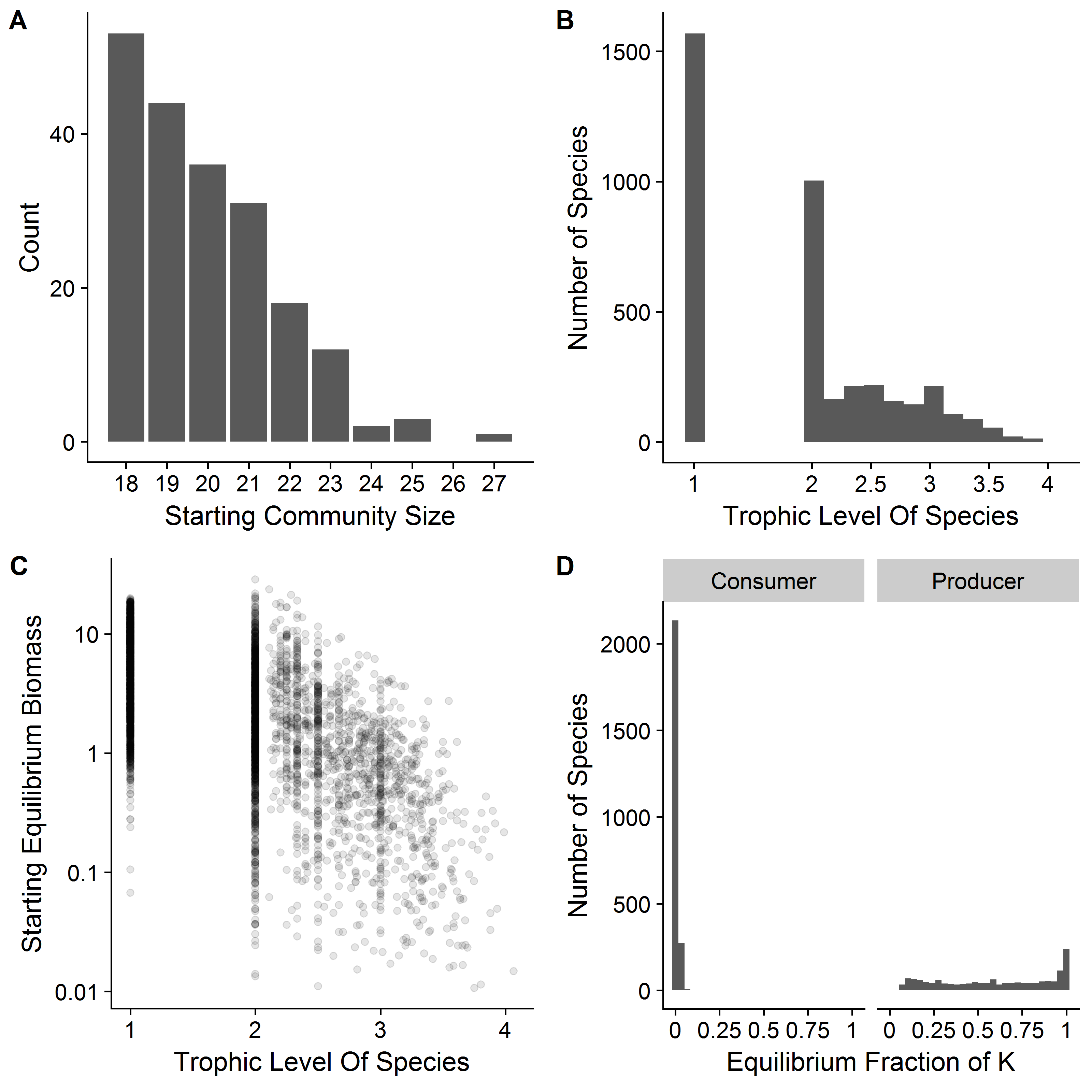


Figure S2. Further details about the properties of the 200 equilibrium communities used in robustness analysis. A: Histogram showing distribution of number of species in each community. Eighteen species was chosen as a lower cut off-point. B: Distribution of 3983 species across trophic levels, as calculated using the unweighted-pathway method. 8 species that had a calculated trophic level above 4 are not shown. C: Relationship between trophic level of a species and its initial equilibrium biomass, showing a general negative relationship. 67 populations has an initial biomass below 0.01 and are not shown for clarity. D: Initial Equilibrium biomass as a fraction of each species carrying capacity. Consumer equilibrium biomass was considerable below carrying capacity in all cases, while producer biomass varied from near capacity to well below.

**3. Derivation of Gompertz Control Parameters**

The basic form of a Gompertz equation that takes a set of parameters $\theta_{ijk}$and an input $x$ is:

$$f\left( \theta_{ijk},x \right) =g\left( a e^{-be^{-cx}}-d \right)$$

In order to maintain the sign of $\mu_{ijk}$ since the sign of the Gompertz function can be negative) we consider:

$$\log_{10} \left( \mu_{ijk} \right)=f\left( \theta_{ijk}, x \right)$$

In our case, we take $x$ to be the magnitude of deviation from the initial population value: $\log_{10} \frac{B_{k}}{B_{k}^{*}}$

The core results that we wish to achieve are:

1. $f\left( \theta_{ijk},0 \right) =0$, when the modifier biomass is at equilibrium, there is no modification effect
2. $f\left( \theta_{ijk},\tau\right) =$, the slope at the point of inflection is α
3. $f^{''}\left( \theta_{ijk},\tau\right)=0$, the point of inflection occurs at $x=\tau$
4. $\left| f\left( \theta_{ijk},\infty\right)-f\left( \theta_{ijk},-\infty\right) \right|=\sigma$, the range of the equation is $\sigma$
5. The function should not, on average, be biased towards increasing or decreasing the strength of the interaction.

The core Gompertz equation ($e^{-be^{-cx}}$) ranges from 0-1, therefore the criterion iv) can be achieved by setting a= σ.

Criterion ii) can be met using the result that $\max\left( \frac{df}{dx} \right)= \frac{gac}{e}$, which can be rearranged to give $c= \frac{\alpha e}{g\sigma}$ .

Criterion iii) can met through solving $f’’\left( x \right)=0$ for x, which is $x= \ln\left( b \right)/c$. Since by definition at this point, $x=\tau,$this can then be rearranged to give $b= e^{c\tau}$. $c$ is given by the previous result.

Since we define x as the magnitude of divergence from the initial population value $B_{k}^{*},$ $\tau$ therefore reflects the population change necessary to reach the threshold point of maximum change. This threshold occurs at: $B_{k}^{threshold} =B_{k}^{*}\times{10}^{\tau}$

Criterion i) can be simply met, since when $x=0, e^{-cx}=1$, we can find $d = a e^{-b}$, where $b$ and $a$ are set from previous results.

Finally to address v) and fix the intrinsic bias of the core equation towards either positive or negative modifications depending on the sign of the modification, we randomly rotate half of the functions around the point (0,0) by setting$g=\left\{ \begin{aligned} -1 & \\ +1 & \end{aligned} \right.$with equal probability.

**4. Criteria for establishing equilibriums**

*Finding of Initial Equilibria.*

Each model generated as described in the main text was initiated with a biomass density of 10. All models are available as R functions in the online code appendix. Models were integrated using the deSolve library within R (Soetaert *et al.* 2010), using the default ‘lsoda’ solver which switches automatically between stiff and non-stiff methods as necessary for the problem. Integration was continued with regular checks whether finishing conditions were met. If the biomass density of a species fell below a density of ${10}^{-10}$ it was removed (its density was set to zero). After each extinction those consumers left without any remaining resources were also set as extinct. If extinctions resulted in the splitting of the community into multiple sub-webs the largest was kept and all the species in the smaller web were set as being extinct.

Models were run for a minimum of 400 time steps, and continued until the community was considered to have reached an equilibrium. This was determined by the mean absolute ratio between the mean population of each species over the previous 200 time unit period and the next was less than 1%. We calculate the mean population density in a block of 200 time steps as:

$$\bar{B_{i}} = \left( \frac{1}{200} \right)\sum_{x=\left( t-200 \right)}^{t} B_{i_{x}}$$

and end the simulation if for all species $i$:

$$0.01> \frac{\left| {\bar{B_{i}}}^{current} - {\bar{B_{i}}}^{previous} \right|}{{\bar{B_{i}}}^{previous}}$$

The density of surviving at the last time point was then used as starting values with a multiple root-finding algorithm from the R package *rootSolve* (Soetaert 2009) using the Newton-Raphson method, with the requirement for positive roots. This approach allowed a faster and accurate identification of true equilibria for slow to converge populations. In a handful of cases this root finding process determined the population biomass equilibrium to be below ${10}^{-10}$. In these cases the model integration was declared a failure.

*Robustness Analysis*

The community models including external mortality and potentially TIMs were generated as described in the main text, with starting values as determined above. Models were integrated to equilibrium as described above, without the root-finding procedure.

*Extinctions*

Every 200 steps any species whose densities at that point were below 10^-4^ had their densities set permanently to zero. While conceivably it is possible that species populations could have returned to a higher level given out non-discrete systems, however this boundary was considerably below the starting population and we considered that any real population would struggle to recover from such a reduction. If consumers were left without any resource species, then those consumers also had their density set to zero and the simulation was run for additional time. This allows for cascades of extinctions up food chains where the lowest levels had been removed, even if the higher tropic level species had slow rates of metabolic loss. If the network became split into multiple trophically disconnected subunits all were retained.

Zero values species biomass could lead to undefined divergences from the initial population $B^{*}$. For the purposes of calculating TIMs, modifier biomasses were assumed to have a minimum value of ${10}^{-10}$ .

*Finishing criteria*

If the mean absolute ratio between the mean population of each species over the previous 200 time unit period and the next was less than 1%, the community was considered to have sufficiently stabilised that any all extinctions will have been observed. If no acceptable equilibrium point had been reached after 100’000 time units or the integrator tolerances were exceeded the run was declared a failure. For statistical tests, only complete pairwise comparisons were used. Failures were dominated by integrator tolerance issues, and were more common with the TIM model:

| **Test** | **Successes** | **%** |
| --- | --- | --- |
| No TIM Model | 3975 | 99.8 |
| Pairwise NTEs | 3897 | 97.8 |
| Full TIMs | 3736 | 93.8 |

**5. Initial response of populations**

To test the initial direction of response to perturbation we examined the direction of movement of populations when the biomasses were set the starting value with the exception of the ‘targeted species’ which was set to 99% of its initial value. This was done across all 200 tested communities, for each species. Of 75960 responses, 61.8% were neutral, 18.6 were negative and 19.5% were positive.

**6. Partition of Modified Trophic Interaction into Trophic and Non-Trophic Components**

For case where i is the focus, j's are the predators of i, l's are the prey of i and k's are the modifier, the original impact term is:

$$\frac{1}{B_{i}}\frac{dB_{i}}{dt}=\ldots\underset{Trophic Term with TIM}{\underbrace{-\mu_{ijk}a_{ij}B_{j}}}$$

First expand $\mu_{ij}$as:

$$\frac{1}{B_{i}}\frac{dB_{i}}{dt}=\ldots- \left( 1 +\left( \mu_{ijk}-1 \right) \right) a_{ij}B_{j}$$

Then partition into a direct trophic effect and a non-trophic effect:

$$\frac{1}{B_{i}}\frac{dB_{i}}{dt}=\ldots\underset{Trophic Term}{-a_{ij}B_{j}}+ \underset{Non-Trophic Term}{a_{ij}B_{j}\left( 1-\mu_{ijk} \right)}$$

Make the assumption that the $B_{j}$ in the non-trophic term is fixed at $B_{j}^{*}$:

$$\frac{1}{B_{i}}\frac{dB_{i}}{dt}=\ldots\underset{Trophic Term}{-a_{ij}B_{j}}+ \underset{Non-Trophic Term}{a_{ij}B_{j}^{*}\left( 1-\mu_{ijk} \right)}$$

This approach is only valid where there is only a single TIM per interaction. Multiple TIMs would require considerable further complex terms to account for synergistic effects of TIMs. A similar process, fixing $B_{i}$ can then be followed to find the impact on the other species.

**7. Distribution of Finishing Populations**

Populations tended to decline during the robustness analysis. The proportional change, calculated as (end - start)/start, across all iterations of the second test was -1 (i.e. extinct) for 27287 cases, between -1 and 0 (implying a decline, but not extinction) in 23000 cases and positive (implying a gain) in 26673 cases. Taking declines and extinctions together, populations reduced in 65.3% of cases. Overall total biomass declined in 3170 out of 3835 (82.7%) test perturbations


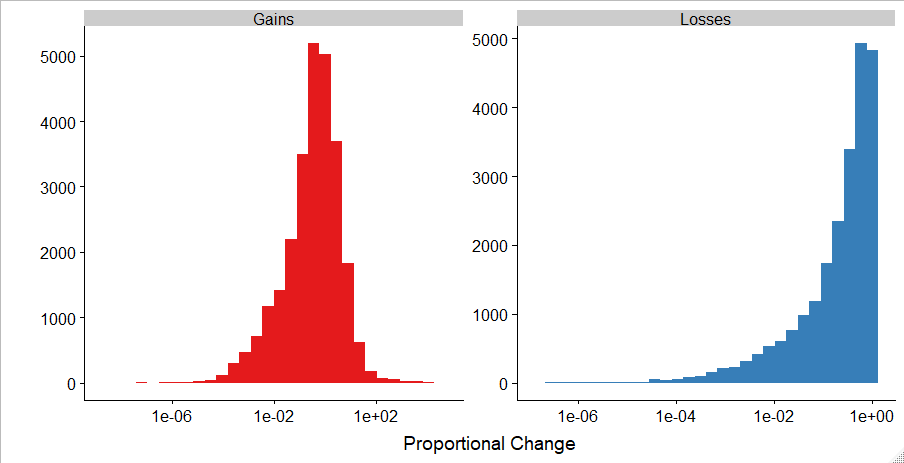


Figure S3. Distributions of proportional change for species that did not go extinct, on a logarithmic scale. Mean $\mathrm{lo}g_{10}$ proportional gain was -0.5, a 30% increase in population density, while mean $\mathrm{lo}g_{10}$ proportional loss (excluding extinctions) was -0.75, a 17% reduction in population density. For reference, if extinct species are included, species that decline, decline on average by 45% reduction.

**References For Supplementary Material**

Levine, S. 1980. Several measures of trophic structure applicable to complex food webs. Journal of Theoretical Biology 83:195–207.

Soetaert, K. 2009. rootSolve: Nonlinear root finding, equilibrium and steady-state analysis of ordinary differential equations.

Soetaert, K., T. Petzoldt, and R. W. Setzer. 2010. Package deSolve : Solving Initial Value Differential Equations in R. Journal Of Statistical Software 33:1–25.
